# Effect of Retinal on *Dictyostelium* Cells During Development

**DOI:** 10.1111/gtc.70037

**Published:** 2025-07-02

**Authors:** Kazuki Akiyama, Shuhei Tsuchihashi, Yusuke V. Morimoto

**Affiliations:** ^1^ Graduate School of Computer Science and Systems Engineering Kyushu Institute of Technology Fukuoka Japan; ^2^ Department of Physics and Information Technology Faculty of Computer Science and Systems Engineering, Kyushu Institute of Technology Fukuoka Japan

**Keywords:** cAMP signal relay, cytoskeleton, *Dictyostelium*, retinal, signal transduction

## Abstract

Retinal plays a key role in light absorption across prokaryotes and eukaryotes, both in rhodopsin and bacteriorhodopsin systems. The multicellular social amoeba *Dictyostelium discoideum* exhibits positive phototaxis. However, retinal binding proteins such as rhodopsin have not been found in the genome of *Dictyostelium* cells. Herein, we microscopically examined the effects of retinal on *Dictyostelium* cells. On adding all‐*trans*‐retinal to the medium, *Dictyostelium* cells retracted their pseudopodia and became rounded. This was unique to retinal among the tested vitamin A variants. Addition of all‐*trans*‐retinal at low concentrations did not cause cell rounding. However, it increased the frequency of cAMP signaling triggered during cell development. Results indicate that retinal acts on an unknown signaling pathway involving the cytoskeleton in *Dictyostelium* cells.

## Graphical abstract

When all‐*trans*‐retinal was added to the medium, *Dictyostelium* cells retracted their pseudopodia and became rounded; however, this rounding was not observed at low concentrations. Instead, low concentrations of all‐*trans*‐retinal increased the frequency of cAMP signaling during cell development.

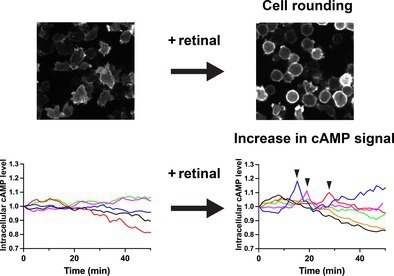

## 1 Introduction

Retinal molecules act as chromophores, forming complexes with opsin proteins such as rhodopsin. This forms the basis of vision in animals. In the human visual system, 11‐*cis*‐retinal absorbs photons and is subsequently converted into all‐*trans*‐retinal (Ernst et al. 2014; Kojima and Sudo 2023; Palczewski 2006; Spudich et al. 2000). This conformational change triggers a signaling cascade involving opsins, which function as G protein‐coupled receptors (GPCRs), ultimately resulting in light perception by the human brain. In contrast, in microbial rhodopsins such as bacteriorhodopsin and channelrhodopsin, retinal is bound in the all‐*trans*‐retinal form and undergoes a conformational change to 13‐*cis*‐retinal upon light absorption, enabling ion transport (Ernst et al. 2014; Kojima and Sudo 2023). Channelrhodopsin is derived from microorganisms but has been utilized in optogenetics to manipulate the membrane potential of mammalian nerve cells (Deisseroth and Hegemann 2017; Jennings and Stuber 2014).

The social amoeba *Dictyostelium discoideum* has been studied as a model organism for cyclic adenosine 3′,5′‐monophosphate (cAMP) signaling via GPCR (Hashimura et al. 2019; Kamimura and Ueda 2021; Loomis 2014; Morimoto 2024; Saran et al. 2002). *Dictyostelium* cells normally grow as unicellular cells, but when starved, cells aggregate and form multicellular bodies through cell‐to‐cell signaling using cAMP (Hashimura et al. 2019; Loomis 2014). Periodic cAMP signaling relays result in coordinated collective migration and the formation of multicellular bodies. As multicellular bodies develop and migrate as a multicellular state called a slug, the oscillatory cAMP signal disappears (Hashimura et al. 2019, 2022). The anterior and posterior parts of the slug differentiate and partition into the prestalk and prespore cells, respectively (Williams 2006). Prespore cells eventually become spores, remain dormant, and germinate into amoebae when the environment is suitable. Prestalk cells differentiate into stalk cells in the fruiting body, undergo cell death, and function to lift the spores. This differentiation into stalk cells, which leads to cell death, is induced by DIF‐1 and c‐di‐GMP (Chen and Schaap 2016; Cornillon et al. 1994; Song et al. 2015).

*Dictyostelium* multicellular slugs exhibit markedly phototaxis (Fisher 1997). The slug senses light in the anterior prestalk cells, especially during tip activation (Fisher and Annesley 2006). A heme protein isolated from the mitochondria has been proposed as a candidate photoreceptor pigment (Poff and Butler 1974; Poff et al. 1974); however, its receptors have not been validated using a molecular genetic approach (Fisher 1997). Light irradiation promotes the synthesis of a Slug Turning Factor (STF), and the slug achieves directional turning toward the light source along the STF gradient formed within it (Fisher et al. 1981). The signaling pathway of phototaxis in slugs is associated with G proteins, the second messengers cAMP, cGMP, IP_3_ and Ca^2+^, protein kinases, and cytoskeletal proteins (Fisher and Annesley 2006; Miura and Siegert 2000). As cell motility is also involved in phototaxis, many signaling pathways contribute to light responses, but the main signaling pathway remains unknown (Miura and Siegert 2000). *D. discoideum* cells tend to assemble under irradiated light, even during the unicellular stage, but this is thought to differ from the phototaxis signaling system of slugs (Häder and Poff 1979). No rhodopsin or bacteriorhodopsin has been found in the genome sequence of *D. discoideum* (Eichinger et al. 2005). However, homology searches alone are insufficient to inform whether *D. discoideum* has any retinal‐binding proteins. For example, heliorhodopsin (Pushkarev et al. 2018), a newly discovered retinal‐binding protein, has low sequence homology to known retinal‐binding proteins. In order to investigate whether *Dictyostelium* cells have a signaling pathway mediated by retinal‐binding proteins, we examined the effects of retinal addition on the behavior of *Dictyostelium* cells.

## 2 Results

### 2.1 Retinal Induces Cell Rounding in *Dictyostelium*

**FIGURE 1:**
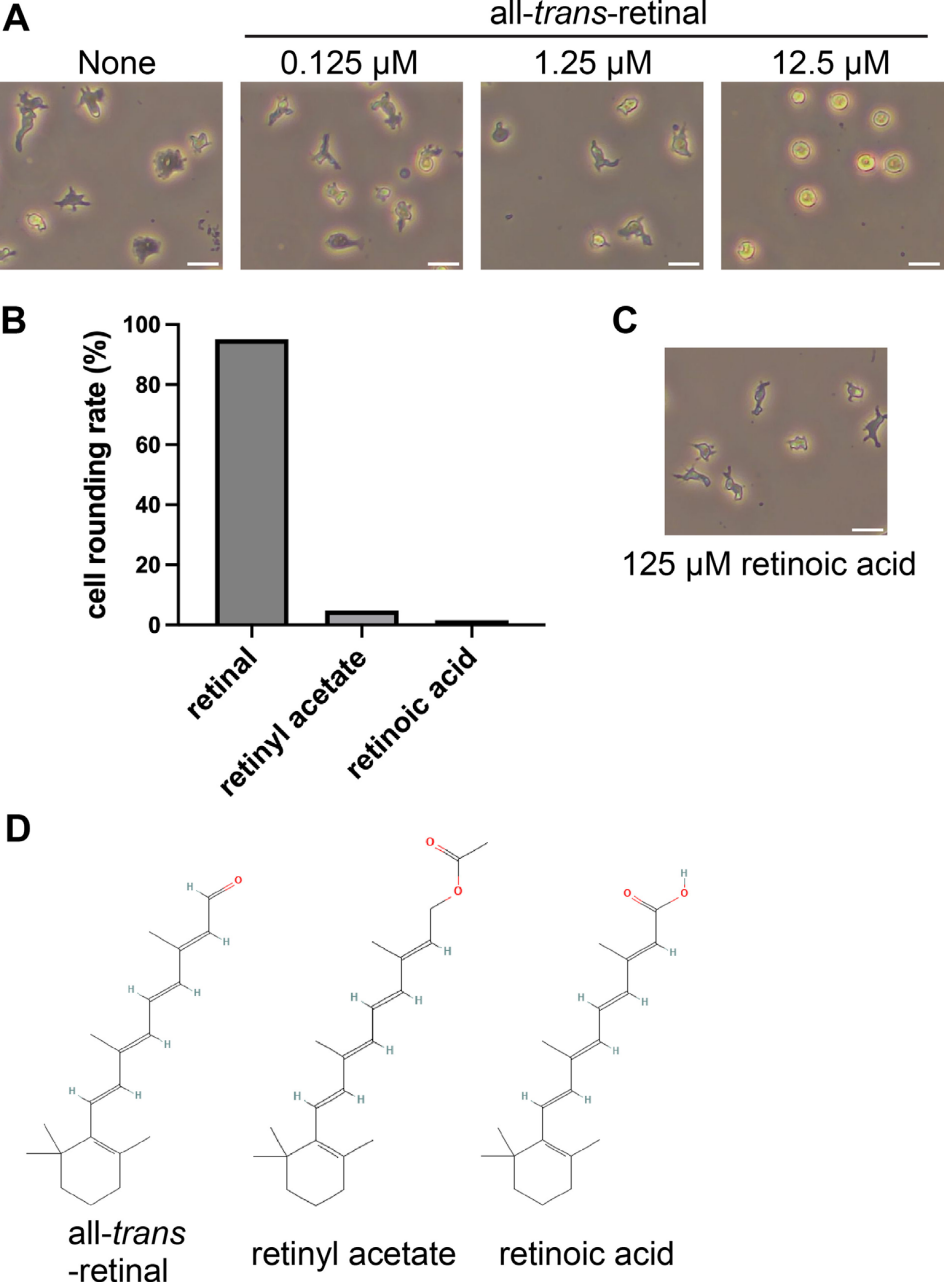
Effects of retinal on *Dictyostelium* AX2 cell morphology. (A) After starvation of AX2 cells in DB for 5 h, the cell morphology was captured under a phase‐contrast microscope 15 min after the addition (at different concentrations) or absence (“None”) of retinal. All‐*trans*‐retinal was added at a final concentration of 0.125, 1.25, or 12.5 μM. (B) The percentage of rounded cells was estimated from the micrographs when different vitamin A variants were added. 12.5 μM all‐*trans*‐retinal, 12.5 μM retinyl acetate, or 12.5 μM retinoic acid were tested. Each value was estimated for > 70 cells. (C) A phase contrast microscopy image of *Dictyostelium* cells with 125 μM retinoic acid. Scale bars indicate 20 μm. (D) Chemical structures of all‐*trans*‐retinal, retinyl acetate, and retinoic acid acquired from PubChem (Kim et al. 2025).

To investigate the effect of retinal on *Dictyostelium* cells, we added a stable variant of retinal, all‐*trans*‐retinal, to the medium containing the cells and observed the effects. When 12.5 μM all‐*trans*‐retinal was added to the medium, the cells contracted their pseudopodia and rounded in shape (Figure 1A). Retinal is the aldehyde form of vitamin A. The alcohol form of vitamin A is called retinol, which when oxidized becomes retinoic acid (Carazo et al. 2021). Retinyl acetate is an acetate ester of retinol. To study the effects of different vitamin A variants, we next tested the effect of retinyl acetate and retinoic acid on the cells. Retinyl acetate and retinoic acid, unlike retinal, did not cause cell rounding (Figure 1B). Higher concentrations of retinoic acid (125 μM) also had no effect on cell morphology (Figure 1C). These results suggest that cell rounding is specifically caused by retinal.

**FIGURE 2:**
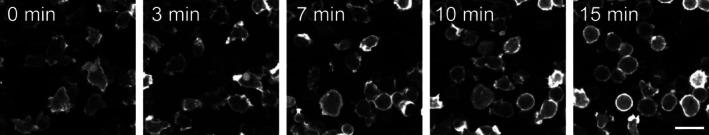
Actin dynamics after the addition of retinal to *Dictyostelium* cells. F‐actin was labeled with LifeAct14‐mScarletI and was observed in AX2 cells. After adding 40 μM all‐*trans*‐retinal, F‐actin dynamics were measured in time‐lapse. Fluorescence images are shown at 0, 3, 7, 10, and 15 min after retinal addition. Scale bar indicates 20 μm.

Cell rounding is caused by inhibition of actin (Arai et al. 2010; Gerisch et al. 2004). Changes in cell shape after the addition of all‐*trans*‐retinal were observed under a fluorescence microscope using LifeAct14‐mScarletI labeled F‐actin, which acts as a cytoskeleton (Figure 2). Approximately 15 min after the addition of retinal, the pseudopodia gradually subsided, and the cells became rounded. These changes suggest that retinal suppresses pseudopodial elongation, resulting in cell rounding (Figure 2, Movie S1).

### 2.2 Effect of Retinal on *Dictyostelium* Cell Assembly During Development

**FIGURE 3:**
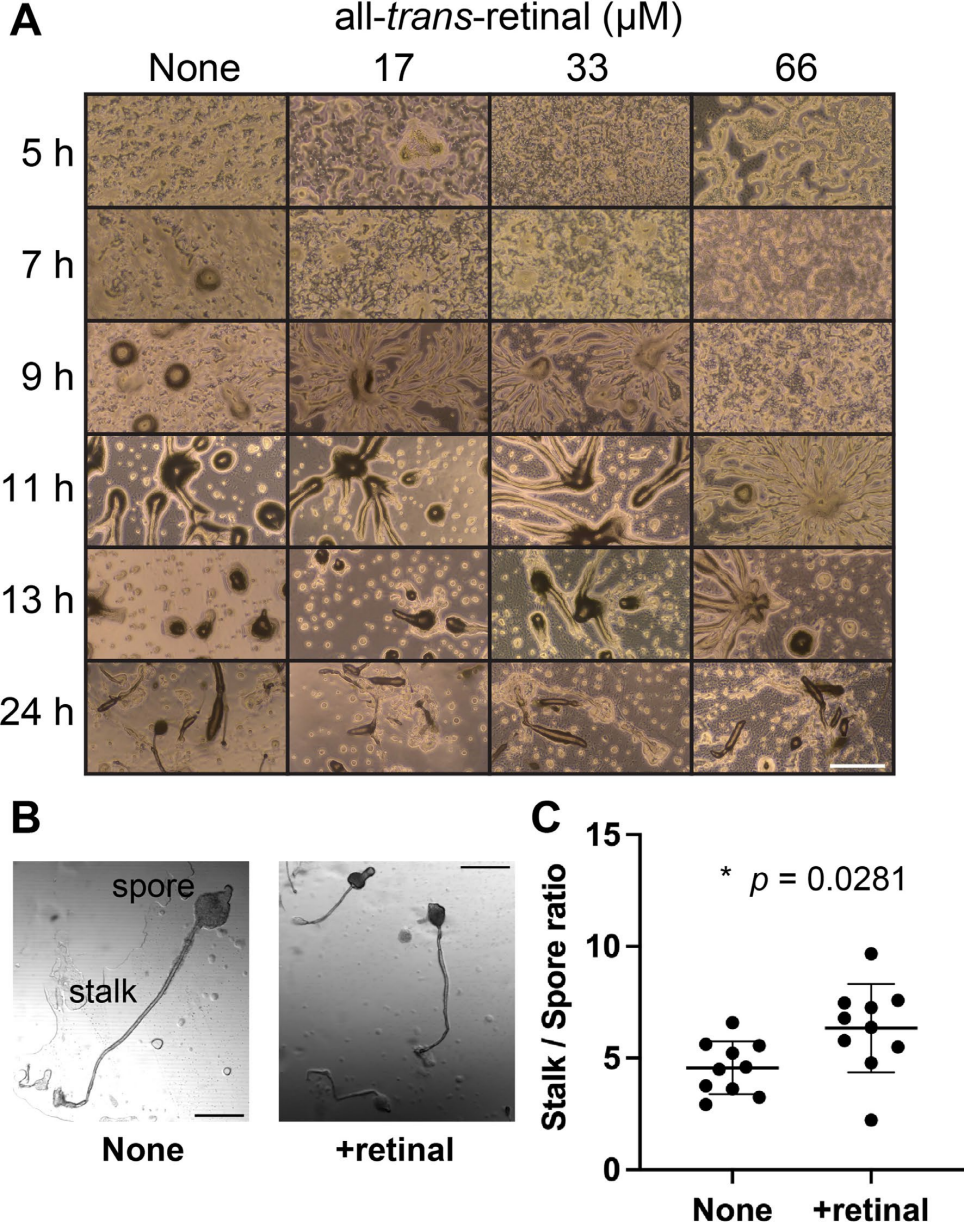
Effects of retinal on *Dictyostelium* cell development. (A) Cells suspended in DB with or without retinal were spread on agar and their developmental process was observed under a microscope. 17, 33, or 66 μM all‐*trans*‐retinal were tested. The time points indicate the time elapsed after spreading the cells onto the agar. Scale bar indicates 500 μm. (B) Representative images of fruiting bodies with and without 33 μM all‐*trans*‐retinal (retinal). Scale bars indicate 200 μm. (C) The ratio of the lengths of the stalk and spore of the fruiting bodies formed on the agar was measured with or without 33 μM all‐*trans*‐retinal. Spore size included both the upper and lower cup regions. The stalk size was the distance from the base of the lower cup to the disk. Each was estimated from 10 fruiting bodies. Statistical analyses were performed using the Welch's *t*‐test.

During the development for fruiting body formation in *Dictyostelium* cells, differentiation into stalk cells is accompanied by cell death, which results in cell rounding (Song et al. 2015). In order to observe a uniform and rapid response to the addition of vitamin A variants, cells were initially examined in solution, as depicted in Figures 1 and 2. However, *Dictyostelium* cells need to be developed on agar to observe complete fruiting body formation (Fey et al. 2007). To investigate whether retinal affects cell development, cells were starved on agar in the presence and absence of retinal (Figure 3A). When cells in the solution with 66 μM all‐*trans*‐retinal were starved on agar, cell development proceeded as normal and fruiting bodies were formed; however, the onset of cell aggregation was slowed and development was markedly delayed. When comparing fruiting bodies with and without 33 μM all‐*trans*‐retinal, those formed in the presence of retinal were generally smaller in overall size (Figure 3B). Although the difference was modest, a statistically significant change was observed in the stalk‐to‐spore ratio in the presence of retinal; the spore mass was smaller and the stalk was relatively longer (Figure 3C). Retinal does not easily penetrate the entire multicellular body when applied in agar and affects the cells at a higher local concentration compared to application in liquids (Figure 1). These results suggest that the addition of retinal may affect cell assembly during development.

### 2.3 Retinal Affects cAMP Signaling in *Dictyostelium* Cells

**FIGURE 4:**
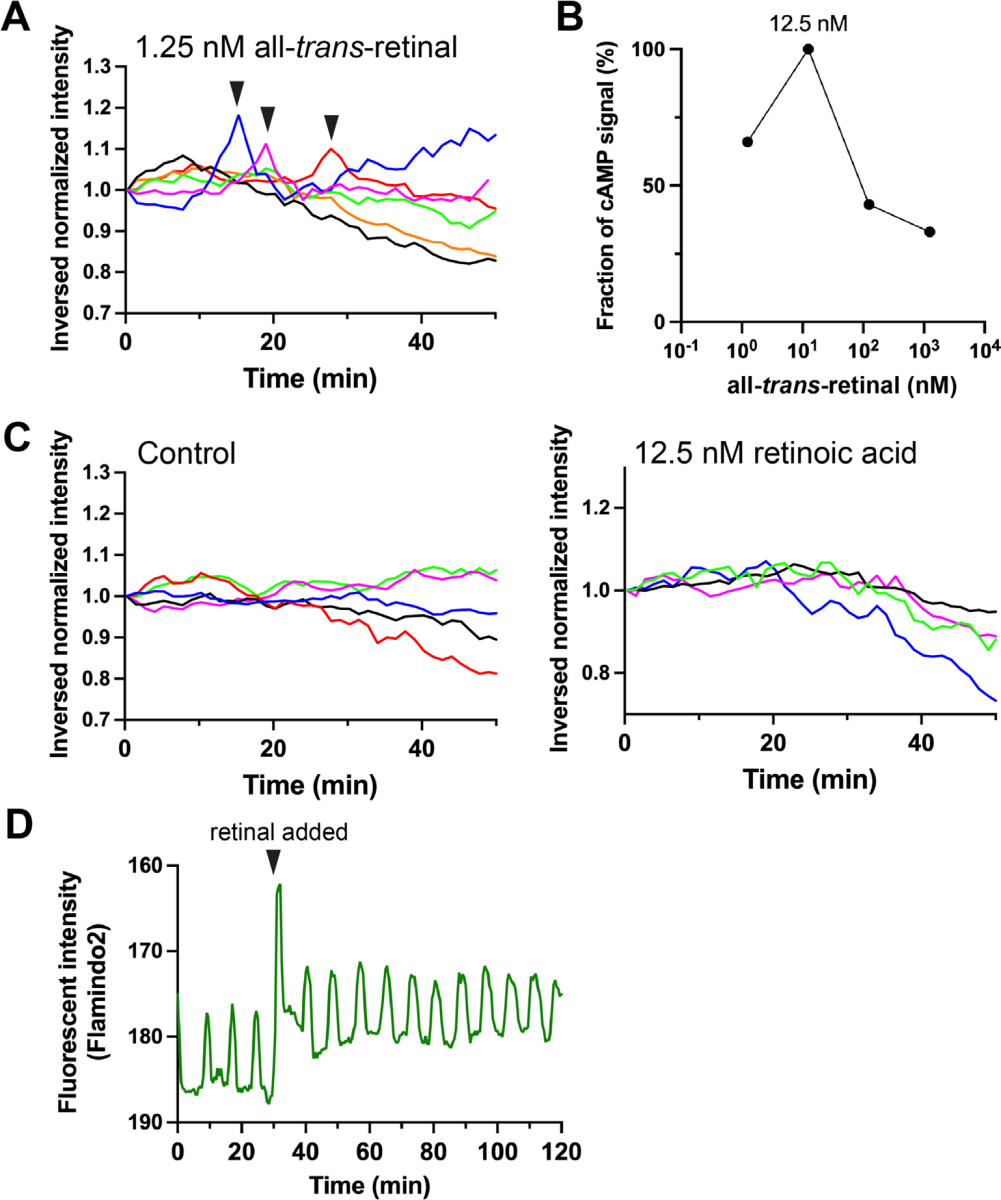
Effects of retinal on cAMP signaling in *Dictyostelium* cells. (A) Starved AX2 cells expressing the cAMP indicator Flamindo2 were observed under a fluorescence microscope for at least 50 min after 1.25 nM all‐*trans*‐retinal was added. Changes in the inverted Flamindo2 fluorescence intensity values in cellular regions throughout the field of view were plotted. Data for each experiment are presented in different colors. The firing points of the cAMP signal are indicated by arrowheads. (B) The frequency of cAMP firing at least once every 50 min at each retinal concentration. 1.25 nM, 12.5 nM, 125 nM, or 1.25 μM all‐*trans*‐retinal were measured. Each value was estimated from more than five data points. (C) Changes in inverted Flamindo2 fluorescence intensities within cells after adding buffer without retinal (left panel) or with 12.5 nM retinoic acid (right panel) are plotted. Data for each experiment are presented in different colors. (D) The effect of retinal addition on cells expressing Flamindo2 in which cAMP signaling relays occur in a periodic manner. 12.5 nM all‐*trans*‐retinal was added 30 min after the start of measurement (arrowhead). The vertical axis is inverted.

To investigate the effect of retinal on cAMP signaling during the cell assembly initiation phase, *Dictyostelium* cells expressing the cAMP probe Flamindo2 (Hashimura et al. 2019; Odaka et al. 2014) were starved, and all‐*trans*‐retinal was added at different concentrations to the buffer containing the cells. Retinal was added 3 h after starvation, at which time the cAMP signal was still low, and the frequency of cAMP signal firing increased. The frequency was highest at 12.5 nM. However, at higher tested concentrations (125 nM and 1.25 μM), the signal frequency decreased (Figure 4B).

To test the effect of the other vitamin A variant, retinoic acid, the buffer solution containing cells was treated with 12.5 nM retinoic acid. However, it did not induce cAMP signaling as retinal did (Figure 4C). Thus, the effects on cAMP signaling also appear to be retinal‐specific, similar to the retinal‐specific effect on the induction of cell rounding. To determine whether retinal addition perturbed the cAMP signaling relay, retinal was added to a population of cells that were developing and producing periodic cAMP signals. The addition of the solution transiently affected the fluorescence observation but did not change the cycle or amplitude of the cAMP signal oscillation before and after retinal addition (Figure 4D).

### 2.4 Effect of Retinal Is Different From That of Cell Death Induction in *Dictyostelium* Cells

Cell death in stalk cells is induced by DIF‐1 and c‐di‐GMP, which act as signaling molecules of stalk cell differentiation (Chen and Schaap 2012; Ide et al. 2023; Thompson and Kay 2000). c‐di‐GMP‐induced cell death is inhibited by *dmtA* ^−^ and *stlB* ^−^ knockouts, which are defective in polyketide synthesis, suggesting that DIF‐1 synthesis is required for cell death (Song et al. 2015). Cell death induced by DIF‐1 is inhibited through different pathways and by knockout of the *iplA* gene (Song et al. 2015). We tested whether the effects of retinal‐induced cell rounding were altered in *dmtA* ^−^, *stlB* ^−^, and *iplA* ^−^ knockout strains (Figure S1). Similar to the wild type, the three mutant strains showed rounding of cells after the addition of 12.5 μM all‐*trans*‐retinal. When cell rounding occurs, cells stop amoebic motility. However, after 5 h, cells treated with retinal grow pseudopodia and begin motility again, progressing to cell assembly (Figure S2). These results suggest that the effect of retinal is different from that of cell death induction.

Since retinal acts as a cofactor for rhodopsin, we tested whether retinal is involved in phototaxis by binding to and interacting with an unknown rhodopsin‐like protein. We observed phototaxis during the multicellular slug stage with and without 1.25 μM all‐*trans*‐retinal, a concentration at which cell rounding does not occur. We found no clear difference in phototaxis in the presence or absence of retinal (Figure S3).

## 3 Discussion

In this study, we examined the effects of retinal addition on the behavior of *Dictyostelium* cells and found that retinal induced cell rounding in *Dictyostelium* (Figures 1 and 2). This effect peaks at a 12.5 μM concentration of retinal and was specific only to retinal among all tested vitamin A variants. Further, we found evidence that retinal may affect *Dictyostelium* cell assembly during development and has an effect on cAMP signaling (Figure 4).

Retinal treatment increased the frequency of cAMP signaling in *Dictyostelium* cells (Figure 4A,B). However, the response did not immediately follow the addition of retinal, varying from 7 to 40 min after addition, suggesting that it more likely promotes development rather than acting directly on the cAMP signaling pathway. This was also suggested by the lack of a perturbing effect on the oscillating cAMP signals (Figure 4D). In contrast, the addition of retinal to agar delayed development (Figure 3A). This may be due to the antagonism between mechanisms promoting development at low concentrations and the induction of cell rounding at high concentrations. Therefore, it is unclear whether the promotion of development and induction of cell rounding act via the same pathway. The underlying molecular pathways need to be identified. In addition, although a stable all‐*trans*‐retinal preparation was used in this study, isomerization can occur under experimental conditions such as light exposure; therefore, comparisons with the corresponding *cis*‐isomers are required.

Through a multicellularization process, *Dictyostelium* cells form fruiting bodies composed of spores and a stalk (Loomis 2014; Morimoto 2024). Cell death, which causes cell rounding via stalk cell differentiation, is induced by DIF‐1 and c‐di‐GMP (Song et al. 2015). However, the retinal‐specific cell rounding observed in this study occurred in *dmtA*, *stlB*, and *iplA* knockouts, as well as in wild‐type cells (Figure S1). Furthermore, the cells began to extend pseudopodia over time after the addition of retinal (Figure S2). These results suggest that retinal‐induced cell rounding proceeds through a signaling pathway different from that of cell death induction.

*Dictyostelium* cells exhibit remarkable phototaxis in multicellular bodies; however, the signaling pathway underlying this behavior remains only partially understood (Fisher 1997; Fisher and Annesley 2006; Flegel et al. 2011; Miura and Siegert 2000). Phototaxis of the slugs shows main peaks at 420 and 440 nm, with secondary peaks at 560 and 610 nm (Poff and Häder 1984). No clear homologs of rhodopsin or bacteriorhodopsin proteins have been identified in the genome (Eichinger et al. 2005). We also performed BLAST homology searches (Boratyn et al. 2013) for rhodopsin, bacteriorhodopsin, and heliorhodopsin against the *D. discoideum* genome, but no clear homologous genes were found in *D. discoideum*. Although a definitive pathway for retinal biosynthesis or metabolism has not been identified in *D. discoideum*, the organism does possess genes that encode phosphatidylinositol transfer protein 2 (PitB), which contains a domain conserved in *Drosophila* retinal‐degeneration B (Swigart et al. 2000), and AU074982, which encodes a retinal pigment‐epithelium protein (Maeda et al. 2003). In addition, *D. discoideum* possesses dgat1 and dgat2, which encode diacylglycerol *O*‐acyltransferases and are involved in retinol metabolism (Du et al. 2014). These genes may indicate previously unrecognized roles for retinal in *D. discoideum*. In fact, the presence of a retinal‐specific cellular response suggests that retinal‐binding proteins are retained in *Dictyostelium*. In this study, retinal addition had no clear effect on phototaxis during the slug stage (Figure S3). It is possible that retinal is used as a metabolite rather than for a photosensitive function in *Dictyostelium*. In this case, retinal may be used with high specificity since no effect was observed with other vitamin A variants (Figures 1 and 4). It is also possible that *Dictyostelium* cells have non‐GPCR‐type retinal‐binding proteins, but they may also have unknown proteins with GPCR‐like structures that cannot be found by homology searches alone, as is the case with heliorhodopsin, which was discovered relatively recently (Pushkarev et al. 2018). If a retinal‐binding protein with a GPCR‐like structure exists in cellular slime molds, it could possibly be revealed in future screening studies.

Vitamin A‐binding proteins, such as rhodopsin and other retinal‐binding proteins, as well as the retinol‐binding protein family, are widely conserved in living organisms (Carazo et al. 2021; Kojima and Sudo 2023; Spudich et al. 2000). Retinoic acid signaling regulates vertebrate development (Cunningham and Duester 2015; Ghyselinck and Duester 2019) and is associated with the actin cytoskeleton (Mukherjee et al. 2009). Studies of vitamin A in *Dictyostelium* have reported that retinol and all‐*trans*‐retinoic acid reduce the activity of ribonuclease P (Papadimou et al. 1998). The potential relationship between the effects of retinal on cells—observed in this study—and ribonuclease may warrant further investigation. Vitamin A metabolism in cellular slime molds is poorly understood, and future studies in this direction may further uncover its mechanisms.

Actin is a cytoskeleton that plays an abortive role in pseudopodia formation and controls cell motility and cell morphology (Lee and Dominguez 2010; Ridley et al. 2003). Inhibitors of actin polymerization, such as latrunculin and cytochalasin, decrease pseudopodial elongation and cause cell rounding (Parent et al. 1998; Peng et al. 2011; Servant et al. 2000). In *Dictyostelium* cells, when 0.5–5 μM of latrunculin A or 1 μM of cytochalasin D is added to the medium, the cells become firmly rounded after about 20 min, while the pseudopodia begin to shorten as early as 30 s after addition (Arai et al. 2010; Parent et al. 1998). In contrast, when retinal was used in this study, cells became rounded after 15 min, but the shortening of the pseudopodia was not visible until around 5 min after addition (Figure 2, Movie S1). This suggests that retinal acts upstream of actin, rather than directly on actin. Although this study dealt with live cells, studies using purified actin and other actin‐related proteins will be required in the future.

Retinal is rarely used outside studies involving rhodopsin‐expressing cells. However, with the advent of optogenetics, channelrhodopsin has been widely adopted as an optogenetic tool (Deisseroth and Hegemann 2017), and retinal is sometimes added to the medium as a cofactor in such experiments (Dawydow et al. 2014). This study provides a reference for effective retinal concentrations in optogenetic applications using *Dictyostelium*. Moreover, the unidentified retinal‐dependent pathway suggested here may be evolutionarily conserved, highlighting the need to consider endogenous retinal effects in optogenetic systems.

## 4 Experimental Procedures

### 4.1 Cell Strains and Culture Conditions

*D. discoideum* strains used in this study are listed in Table S1. Cells were grown axenically in HL5 medium including glucose (Formedium, UK) in culture dishes at 21°C. Transformants were maintained at 10 μg/mL G418 (Fujifilm Wako, Japan) or 10 μg/mL blasticidin S (Fujifilm Wako, Japan).

### 4.2 Plasmid Construction and Genetic Manipulation

The plasmids used in this study are listed in Table S2. pDM326_LifeAct14‐mScarletI was constructed by inserting a fragment of the red fluorescent protein mScarletI (Bindels et al. 2017) with the LifeAct‐14 sequence added to the N‐terminus at the *Bgl*II and *Spe*I sites of pDM326 (Veltman et al. 2009) using primers. LifeAct‐14 is a peptide that functions as an F‐actin‐specific marker with a similar affinity to LifeAct, but with reduced inhibition of cellular function compared to LifeAct (Bhaskar et al. 2023; Kumari et al. 2020). The wild‐type AX2 strain was transformed by electroporation using a MicroPulser (Bio‐Rad, USA) and the transformants were selected with G418 or blasticidin S (Gaudet et al. 2007).

### 4.3 Observation of Cell Morphology and Dynamics During Development

To induce development upon starvation, cells grown in HL5 medium to ~1 × 10^7^ cells/mL were harvested and washed three times with developmental buffer (DB: 5 mM Na_2_HPO_4_, 5 mM KH_2_PO_4_, 2 mM MgCl_2_, 0.2 mM CaCl_2_, pH 6.5). The medium was then replaced with DB, and the cell suspension was diluted to 1 × 10^5^ cells/mL. After starvation in DB for 5 h, cell morphology was observed with and without the addition either of all‐*trans*‐retinal (Fujifilm Wako, Japan), all‐*trans*‐retinoic acid (Fujifilm Wako, Japan), or retinyl acetate (Sigma‐Aldrich, USA). Multicellular body formation was observed by placing AX2 cells suspended at 1 × 10^7^ cells/mL in DB with or without all‐*trans*‐retinal on 1.5% agar (Bacto Agar, BD, USA). Cell morphology and developmental processes were observed using a phase‐contrast microscope (CKX53, Olympus, Japan) with ×20 objective (UPLFN20XIPC, Olympus, Japan) and a ×4 objective (UPLFN4XIPC, Olympus, Japan), respectively. The images were acquired using a CMOS camera (ATZ, Kenis, Japan).

### 4.4 Fluorescence Image Acquisition and Analysis

Cells were observed at 22°C. Fluorescence images of Flamindo2 were acquired using an inverted fluorescence microscope (ECLIPSE Ti2, Nikon, Japan) with an objective lens (PlanFluor ×20, Nikon, Japan), a fluorescence filter set (GFP‐B, Nikon, Japan), and an sCMOS camera (Prime, Photometrics, USA). Confocal images of LifeAct14‐mScarletI were taken using an inverted microscope (IX83, Olympus, Japan) equipped with a CSU‐W1 confocal scanner unit (Yokogawa, Japan), a 555 nm laser diode illuminator (LDI, 89 NORTH, USA), an sCMOS camera (Prime 95B, Photometrics, USA), and objectives (UPLSAPO ×20×/0.75 NA, Olympus, Japan). To avoid the effects of excess light, observations were made in a dark room. All images were processed and analyzed using Fiji (Schindelin et al. 2012). In the time‐lapse imaging of Flamindo2, the fluorescence intensities of Flamindo2 were normalized with values at *t* = 0, and the decay due to fluorescence bleaching was corrected by exponential function fitting. As the fluorescence intensity of Flamindo2 decreases with increasing intracellular cAMP concentration (Odaka et al. 2014), inverted fluorescence intensity values were plotted. The occurrence of cAMP signaling was determined based on both the fluorescence intensity waveform data and the time‐lapse images.

### 4.5 Observation of Phototaxis in Multicellular Slugs

The slugs were allowed to develop on agar, and phototaxis was observed under an inverted microscope (IX73, Olympus, Japan) with an objective lens (UPlanSApo ×4, Olympus, Japan) and an sCMOS camera (Zyla4.2, Andor, UK). Halogen lamp light for observation was filtered with a short‐wave cutoff filter (SCF‐50S‐60R, OptoSigma, Japan). A 35 mm plastic dish containing the slugs on agar was illuminated from one side by a white LED light source (ca. 8.8 mW, STA‐B2, Shimadzu, Japan). The angle of the arc made by the tip of the slug relative to the light source, from the start of measurement to 100 min later, was calculated.

### 4.6 Statistical Analyses

All statistical analyses were performed using the Welch's *t*‐test in Prism 9 (GraphPad, USA).

## Author Contributions

**Kazuki Akiyama:** data acquisition, data analysis. **Shuhei Tsuchihashi:** data acquisition, data analysis. **Yusuke V. Morimoto:** conceptualization, data acquisition, data analysis, supervision, writing – original draft, funding acquisition. All the authors have reviewed the manuscript and agree to its submission.

## Ethics Statement

The authors have nothing to report.

## Conflicts of Interest

The authors declare no conflicts of interest.

## Supporting information


**Data S1.** Supporting Information.

**Movie S1.** Actin dynamics after retinal addition to *Dictyostelium* cells. F‐actin was labeled with LifeAct14‐mScarletI and was observed in AX2 cells. After adding 40 μM all‐*trans*‐retinal, F‐actin dynamics were measured in time‐lapse for 20 min. Scale bar indicates 50 μm.

## Data Availability

The data that support the findings of this study are available from the corresponding author upon reasonable request.
