# Supplementary material for: Effect of Retinal on Dictyostelium Cells During Development
Source: Genes Cells. 2025 Jul 2;30(4):e70037. doi: 10.1111/gtc.70037 (PMC12221695; doi:10.1111/gtc.70037)
Supplement: Supplementary file 1 — Data S1. Supporting Information. [file GTC-30-0-s002.pdf]

Supporting information

Supplementary Figures

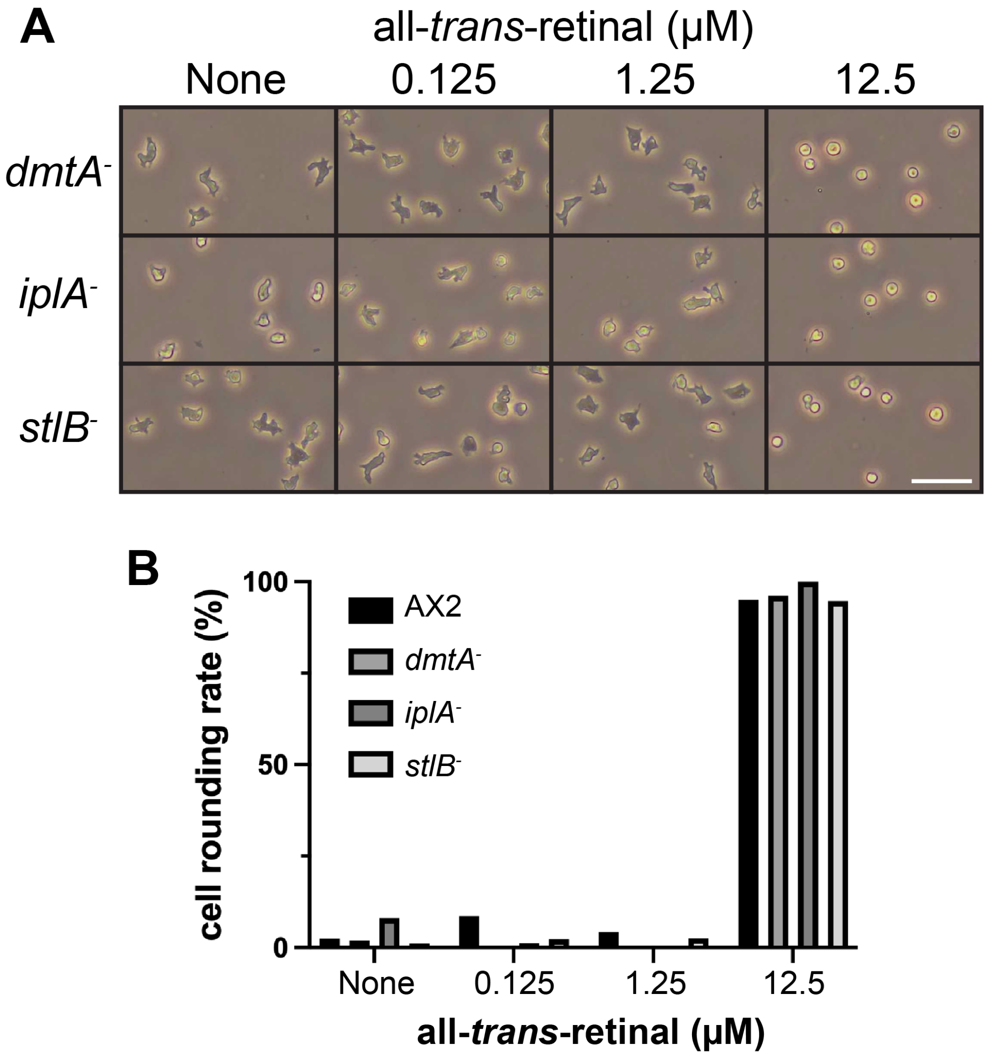

Figure S1

Effect of retinal on the morphology of *Dictyostelium* mutant cells. (A) After starvation of *dmtA*⁻, *iplA*⁻, and *stlB*⁻ cells in DB for 5 h, cell morphologies were captured under a phase contrast microscope 15 min after the addition (at different concentrations) or absence ("None") of retinal. All-*trans*-retinal was added at final concentrations of 0.125, 1.25, or 12.5 μM. Scale bar indicates 50 μm. (B) The percentage of rounded cells was estimated from micrographs with and without retinal. Each bar on the graph was estimated based on data from more than 55 cells.

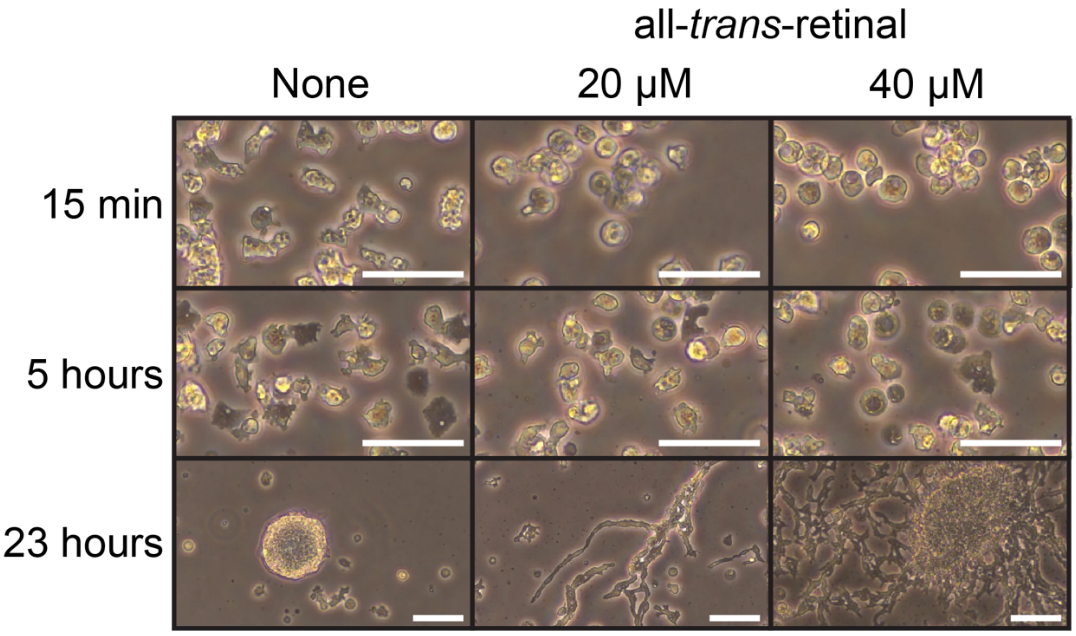

Figure S2
Effect of retinal on development in buffer solution in *Dictyostelium* AX2 cells. (A) After starvation of AX2 cells in DB for 5 h, the cells were imaged under a phase-contrast microscope 15 min, 5 h, and 23 h after the addition (at different concentrations) or absence (“None”) of retinal. All-*trans*-retinal was added at final concentrations of 20 or 40 μM. Scale bars indicate 50 μm.

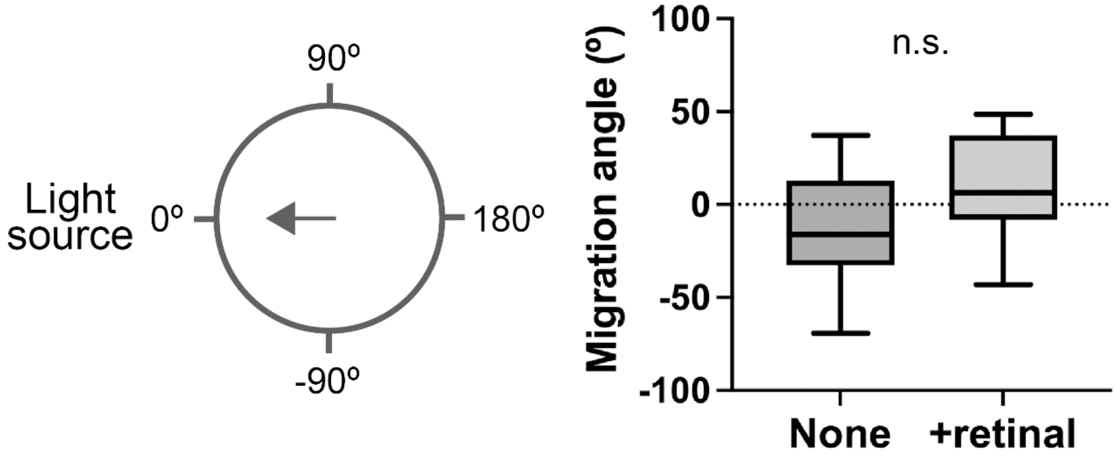

Figure S3
Effect of retinal on phototaxis in *Dictyostelium* AX2 slugs. The slug multicellular bodies on agar were illuminated from one direction, and their travel in the direction of the light source was measured with or without 1.25 μM all-*trans*-retinal (retinal). The cells were observed under a microscope and the angle to the light source in the direction they traveled was estimated at 100 min. Each measured 15 slugs. Statistical analyses were performed using the Welch's *t*-test.

Supplementary Tables

Table S1.

Strain name	Characteristics	Background	Source or reference
AX2	wild type	AX2	Lab stock
iplA^{-}	*iplA* null (bsR)	AX2	Traynor *et al.*, 2000<br>NBRP-nenkin (ID: S00009)
dmtA^{-}	*dmtA* null (bsR)	AX2	Thompson *et al.*, 2000<br>NBRP-nenkin (ID: S00070)
stlB^{-}	*stlB* null (bsR)	AX2	Austin *et al.*, 2006<br>NBRP-nenkin (ID: S90270)
Flamindo2 / AX2	(neoR)	AX2	Hashimura *et al.*, 2019<br>NBRP-nenkin (ID: S90789)
LifeAct14-mScarletI / AX2	(bsR)	AX2	This study

Table S2.

Plasmid name	Characteristics	Backbone	Source or reference
pHK12neo_Dd-Flamindo2	Flamindo2	pHK12neo (neoR)	Hashimura *et al.*, 2019<br>NBRP-nenkin (ID: G90480)
pDM326_LifeAct14-mScarletI	LifeAct14-mScarletI	pDM326 (bsR)	This study

Legend of supplementary movie

Movie S1

Actin dynamics after retinal addition to *Dictyostelium* cells. F-actin was labeled with LifeAct14-mScarletI and was observed in AX2 cells. After adding 40 μM all-*trans*-retinal, F-actin dynamics were measured in time-lapse for 20 min. Scale bar indicates 50 μm.
